# Factors based on optical coherence tomography correlated with vision impairment in diabetic patients

**DOI:** 10.1038/s41598-021-82334-w

**Published:** 2021-02-04

**Authors:** Hiroaki Endo, Satoru Kase, Hikari Tanaka, Mitsuo Takahashi, Satoshi Katsuta, Yasuo Suzuki, Minako Fujii, Susumu Ishida, Manabu Kase

**Affiliations:** 1 Department of Ophthalmology, Teine Keijinkai Hospital, Sapporo, Japan; 2 Department of Ophthalmology, Faculty of Medicine and Graduate School of Medicine, Hokkaido University, N15 W7, Kita-ku, Sapporo, 060-8638 Japan

**Keywords:** Optical materials and structures, Retinal diseases

## Abstract

The aim of this study is to evaluate the relationship between retinal structures and visual acuity in diabetic patients using optical coherence tomography (OCT) and OCT angiography (OCTA). This study was a retrospective observational study conducted at a single medical center in Japan. Evaluation of retinal images was analyzed using spectral domain OCT. Twelve factors including central retinal thickness, length of disorganization of retinal inner layer (DRIL), number of inner hyperreflective foci, number of outer hyperreflective foci, height of intraretinal fluid, height of subretinal fluid, length of external limiting membrane disruption, length of external ellipsoid zone (EZ) disruption, vessel density of superficial capillary plexus (SCP), foveal avascular zone (FAZ) area, and FAZ circularity were analyzed based on OCT/OCTA findings. Multivariate analysis was used to investigate the OCT-based factors that could be correlated with poor visual acuity in treatment-naïve diabetic eyes. A total of 183 eyes of 123 diabetic patients with type 2 diabetes (mean age 61.9 ± 12.3 years, 66 men and 57 women) and 62 eyes of 55 control subjects (mean age 64.4 ± 12.5 years, 15 men and 40 women) was enrolled in this study. Multiple regression analysis showed that OCT-based factors correlated with visual acuity were length of DRIL (β = 0.24, *P* < 0.01), length of EZ disruption (β = 0.35, *P* < 0.001), and FAZ circularity (β =  − 0.14, *P* < 0.05). The other factors showed no significant correlation. In conclusion, the length of DRIL, length of EZ disruption, and FAZ circularity measured by OCT were identified as related factors for visual impairment in treatment-naïve diabetic eyes.

## Introduction

The global prevalence of diabetes mellitus (DM) is estimated to have affected 415 million people in 2015, and is expected to involve 624 million by 2040^1^. Diabetic retinopathy (DR) is the most common ocular complication, which is asymptomatic at an early stage, but progression of the disease leads to severe vision loss^2^. DR is the leading cause of blindness in working-age adults worldwide and is estimated to affect one-third population with diabetes^3^. Diagnosis of DR relies on the detection of microvascular lesions, which is divided into two stages based on their clinical findings: nonproliferative diabetic retinopathy (NPDR) and proliferative diabetic retinopathy (PDR)^4^. The main vision-threatening complication of DR is diabetic maculopathy, including diabetic macular edema (DME)^5^ and diabetic macular ischemia (DMI)^6^, and the presence of vitreous hemorrhage, traction retinal detachment^7^ or chronic inflammation within the proliferative membrane^8^ in PDR. Although treatment of DR remains challenging, timely interventions in eyes at a risk for DR progression have been mandatory for preventing visual impairment^9^.

Optical coherence tomography (OCT) is a non-invasive and fast imaging modality that offers imaging of cross-sectional structures of the retina using low coherence interferometry and captures high-resolution two-dimensional images from scattered light from different layers of the retina^10^. This technique has contributed to the detection and monitoring of DME^11^ and DMI^12^. Several studies have shown that configurations of ellipsoid zone (EZ) are associated with visual function following treatments for DME^13–15^. OCT angiography (OCTA) is an evolutional development of conventional OCT that detects movements of red blood cells and blood flow contrast, visualizing retinal and choroidal microvasculature without the need for dye injection^16^.

In fact, repeated scans at the same location can detect the changes in OCT reflectance signals from the flow through the blood vessels^17^. Recent studies analyzing the role of OCTA in DR have illustrated some quantitative OCTA metrics as linked to the severity of DR and DME^18^. Among the quantitative metrics, vascular density^19^, vascular diameter index^20^, total length of vessels^21^, vascular architecture and branching^22^, and area of the foveal avascular zone (FAZ)^23^ were reliable OCTA-based vascular factors. Thus, OCT and OCTA may help quantify various retinal pathologies such as neurodegeneration, retinal edema, and capillary dropout (reduced vessel density) in DR, thereby assessing their relevance to visual function. However, it has not been fully elucidated which related factors based on OCT(A) findings are well correlated with visual acuity in treatment-naïve DM patients. The purpose of this study is to evaluate the relationship between various retinal structures and visual acuity in DM patients at various stages of DR using OCT and OCTA.

## Results

### Clinical characteristics of control and diabetic patients

A total of 183 eyes of 123 diabetic patients with type 2 DM (mean age 61.9 ± 12.3 years, 66 men and 57 women) and 62 eyes of 55 control subjects (mean age 64.4 ± 12.5 years, 15 men and 40 women) satisfying the inclusion criteria was enrolled in this study. Table 1 shows the clinical characteristics of the subject patients. The DM group (n = 183) was classified into three groups, no DR (NDR) (n = 92), NPDR (n = 67), and PDR (n = 24). There were no significant differences in age, intraocular pressure, axial length, duration of diabetes, estimated glomerular filtration rate (eGFR), frequency of hypertension (HT) and dyslipidemia (DL) in the DR groups compared to the control group. In contrast, the more severe diabetic retinopathy had significantly lower visual acuity (*P* < 0.05) and higher hemoglobin A1c (HbA1c), systolic blood pressure (SBP) and diastolic blood pressure (DBP) (*P* < 0.05). The total cholesterol in the control group was significantly higher than that in the NDR group (*P* < 0.01).

**Table 1:** Clinical characteristics in subjects examined in this study.

Variable	Control eyes (n = 62)	Stage of DR			
		NDR (n = 92)	NPDR (n = 67)	PDR (n = 24)	*P* value
Age (years)	64.4 ± 12.5	62.2 ± 13.0	63.7 ± 9.3	55.2 ± 14.2	0.06
BCVA (logMAR)	− 0.02 ± 0.12	− 0.02 ± 0.11	0.09 ± 0.23^ad^	0.31 ± 0.37^bde^	< 0.001
IOP (mmHg)	14.5 ± 2.8	15.5 ± 3.3	14.9 ± 2.7	14.5 ± 2.9	0.20
AL (mm)	23.70 ± 1.15	23.59 ± 0.91	23.66 ± 0.85	23.39 ± 1.32	0.86
Diabetes duration (years)	–	10.9 ± 11.1	12.8 ± 9.2	12.2 ± 7.4	0.17
HbA1c (%)	5.6 ± 0.2	7.7 ± 2.2^b^	8.3 ± 2.2^b^	9.4 ± 2.3^bd^	< 0.001
HT (%)	35.5	53.3	55.2	41.7	0.51
SBP (mmHg)	122 ± 12	130 ± 17^a^	144 ± 21^bd^	142 ± 31^a^	< 0.001
DBP (mmHg)	71 ± 10	77 ± 12^a^	82 ± 8^bc^	83 ± 20^a^	< 0.001
eGFR (ml/min/1.73 m^2^)	71 ± 12	77 ± 22	67 ± 28	79 ± 35	0.12
DL (%)	19.4	36.7	48.4	54.2	0.18
TC (mg/dl)	212 ± 35^d^	192 ± 36	208 ± 86	224 ± 76	< 0.01

DR, diabetic retinopathy; NDR, no DR; NPDR, no proliferative DR; PDR, proliferative DR; BCVA, best corrected visual acuity; logMAR, logarithm of the minimum angle of resolution; IOP, intraocular pressure; AL, axial length; HbA1c, hemoglobin A1c; HT, hypertension; SBP, systolic blood pressure; DBP; eGFR, estimated glomerular filtration rate; diastolic blood pressure; TC, total cholesterol.

^a^*P* < 0.05 versus control.

^b^*P* < 0.01 versus control.

^c^*P* < 0.05 versus NDR.

^d^*P* < 0.01 versus NDR.

^e^*P* < 0.05 versus NPDR.

### Comparison between research groups on OCT-based factors

This study examined the differences in OCT-based factors between control eyes and all diabetic eyes classified according to DR stages (Table 2). Central retinal thickness (CRT), length of disorganization of the retinal inner layers (DRIL), number of hyperreflective foci (HF), height of intraretinal fluid (IRF), height of subretinal fluid (SRF), length of external limiting membrane (ELM) disruption, length of EZ disruption, vessel density (VD) of superficial capillary plexus (SCP), and FAZ area were significantly large (*P* < 0.05), and FAZ circularity was significantly small (*P* < 0.01) as the severity of disease increased.

**Table 2:** Comparison of OCT-based factors in control eyes and at different stages of diabetic retinopathy.

Variable	Control eyes (n = 62)	Stage of DR			
		NDR (n = 92)	NPDR (n = 67)	PDR (n = 24)	*P* value
**OCT B and C-scan metrics**					
CRT in central subfield by ETDRS grid (μm)	248 ± 17	255 ± 23	290 ± 73^bd^	351 ± 131^bd^	< 0.001
Frequency of DRIL (%)	0.0	0.0	16.4	41.7	< 0.001
Length of DRIL (μm)	0.0	0.0	123 ± 309^bd^	330 ± 487^bd^	< 0.001
Frequency of HF in total retinal layer (%)	11.3	26.1	56.7	75.0	< 0.001
Number of HF in total retinal layer (n)	0.2 ± 0.6	0.5 ± 1.4	2.4 ± 4.6^bd^	8.0 ± 10.3^bde^	< 0.001
Frequency of HF in inner retinal layer (%)	11.3	22.8	55.2	70.8	< 0.001
Number of HF in inner retinal layer (n)	0.2 ± 0.6	0.4 ± 0.9	1.8 ± 3.0^bd^	4.8 ± 6.0^bd^	< 0.001
Frequency of HF in outer retinal layer (%)	0.0	3.3	17.9	62.5	< 0.005
Number of HF in outer retinal layer (n)	0.0	0.0 ± 0.2	0.6 ± 1.8^bd^	3.2 ± 5.0^bdf^	< 0.001
Frequency of IRF (%)	0.0	0.0	28.4	41.7	< 0.001
Height of IRF (μm)	0.0	0.0	47 ± 99^bd^	99 ± 157^bd^	< 0.001
Frequency of SRF (%)	0.0	0.0	9.0	20.8	< 0.001
Height of SRF (μm)	0.0	0.0	6 ± 24^c^	29 ± 65^bd^	< 0.001
Frequency of ELM disruption (%)	0.0	0.0	4.5	8.3	0.07
Length of ELM disruption (μm)	0.0	0.0	39 ± 206	97 ± 337^c^	< 0.05
Frequency of EZ disruption (%)	0.0	0.0	10.4	54.2	< 0.01
Length of EZ disruption (μm)	0.0	0.0	80 ± 257^ad^	575 ± 602^bdf^	< 0.001
**OCTA metrics**					
VD of SCP in central subfield by ETDRS grid (%)	19.3 ± 6.2	21.0 ± 5.9	18.8 ± 6.7	24.8 ± 8.8^ae^	< 0.01
FAZ area (mm^2^)	0.31 ± 0.09	0.28 ± 0.10	0.32 ± 0.13	0.38 ± 0.27	0.06
FAZ circularity	0.72 ± 0.08	0.71 ± 0.08	0.63 ± 0.11^bd^	0.58 ± 0.15^bd^	< 0.001

OCT, optical coherence tomography; DR, diabetic retinopathy; NDR, no DR; NPDR, no proliferative DR; PDR, proliferative DR; CRT, central retinal thickness; ETDRS, Early Treatment of Diabetic Retinopathy Study; DRIL, disorganization of retinal inner layer; HF, hyperreflective foci; IRF, intraretinal fluid; SRF, subretinal fluid; ELM, external limiting membrane; EZ, ellipsoid zone; OCTA, OCT angiography; VD, vessel density; SCP, superficial capillary plexus; FAZ, foveal avascular zone.

^a^, *P* < 0.05 versus control; ^b^, *P* < 0.01 versus control, ^c^, *P* < 0.05 versus NDR; ^d^, *P* < 0.01 versus NDR; ^e^, *P* < 0.05 versus NPDR; ^f^, *P* < 0.01 versus NPDR.

### Correlation between OCT-based factors and visual acuity in diabetic eyes

In this study, multivariate analysis (multiple regression analysis) was performed to evaluate the effects of various OCT-based factors on visual acuity in diabetic eyes (Table 3). Multivariate analysis showed that length of DRIL (β = 0.24, *P* < 0.01), length of EZ disruption (β = 0.35, *P* < 0.001) and FAZ circularity (β =  − 0.14, *P* < 0.05) were significant factors affecting visual acuity. There was no significant association between the other factors and visual acuity. As a result of multivariate analyses, this study further examined the correlation between each factor of OCT images related to visual acuity in diabetic eyes (Table 4). There was a significant positive correlation between length of DRIL and length of EZ disruption (*r* = 0.71, *P* < 0.01). FAZ circularity had a significant negative correlation with length of DRIL and length of EZ disruption (*r* =  − 0.33 and − 0.31, both *P* < 0.01).

**Table 3:** Multiple regression analysis of the correlation between OCT-based factors and visual acuity.

Variable	Unstandardized coefficients		Standardized coefficients		
	*Β*	Standard error	β	*t*	*P* value
**OCT B and C-scan metrics**					
CRT in central subfield by ETDRS grid	0.0002	0.0004	0.0679	0.4810	0.6311
Length of DRIL	0.0002	0.0001	0.2262	2.7867	< 0.01
Number of HF in inner retinal layer	0.0067	0.0065	0.0925	1.0247	0.3070
Number of HF in outer retinal layer	− 0.0075	0.0094	− 0.0753	− 0.7951	0.4277
Height of IRF	0.0002	0.0003	0.0736	0.6683	0.5048
Height of SRF	0.0002	0.0008	0.0271	0.2888	0.7731
Length of ELM disruption	0.0002	0.0001	0.1257	1.6305	0.1048
Length of EZ disruption	0.0002	0.0001	0.3131	3.9448	< 0.001
**OCTA metrics**					
VD of SCP in central subfield by ETDRS grid	− 0.0036	0.0021	− 0.1061	− 1.6958	0.0917
FAZ area	0.1088	0.0930	0.0682	1.1696	0.2438
FAZ circularity	− 0.3258	0.1152	− 0.1575	− 2.8276	< 0.01

OCT, optical coherence tomography; DM, diabetes mellitus, CRT, central retinal thickness; ETDRS, Early Treatment of Diabetic Retinopathy Study; DRIL, disorganization of retinal inner layer; HF, hyperreflective foci; IRF, intraretinal fluid; SRF, subretinal fluid; ELM, external limiting membrane; EZ, ellipsoid zone; OCTA, OCT angiography; VD, vessel density; SCP, superficial capillary plexus; FAZ, foveal avascular zone.

**Table 4:** Correlation between length of DRIL, length of EZ disruption, and FAZ circularity in diabetic eyes.

Variables (n = 183)	1. Length of DRIL	2. Length of EZ disruption	3. FAZ circularity
1. Length of DRIL	–	0.71**	− 0.33**
2. Length of EZ disruption	0.71**	–	− 0.31**
3. FAZ circularity	− 0.33**	− 0.31**	–

DRIL, disorganization of retinal inner layer; EZ, ellipsoid zone; FAZ, foveal avascular zone.

**P < 0.01.

## Discussion

In this study, we investigated the relationship between quantitative retinal structural abnormalities and visual acuity in patients with type 2 DM by applying OCT and OCTA, and found that length of DRIL, length of EZ disruption, and FAZ circularity were independent factors involving visual acuities.

### Significance of OCT-based factors affecting visual acuity

#### DRIL

DRIL was characterized by unidentifiable boundaries of the ganglion cell layer (GCL)—inner plexiform layer (IPL) complex, inner nuclear layer (INL), and outer plexiform layer (OPL), correlated with worse visual acuity in DME, and the changes in DRIL predicted subsequent visual outcome^24,25^. Ocular disorders caused by retinal vascular dysfunction are likely to complicate disruption of the blood-retinal barrier (BRB), followed by extracellular fluid accumulation in the intraretinal or subretinal space^26^. Although the mechanism of DRIL formation is unknown, it is hypothesized that DM-induced microvascular damages inside the retina may represent structural deformation identified as DRIL based on OCT images^27^. In addition, the PDR may form a fibrovascular membrane that creates traction on the macula. Therefore, multiple mechanisms associated with vascular abnormality and mechanical stress may be involved in DRIL formation. This can lead to deformation or breakage of the synaptic junction between photoreceptors and retinal ganglion cells. Furthermore, although a histological observation of DRIL has not been demonstrated, DRIL reflects damage of various cells such as Müller cells, bipolar cells, horizontal cells, and amacrine cells, and is believed to affect the visual outcome of diabetic eyes^25^. Our results, showing the correlation with the length of DRIL and visual acuity, support the findings of previous reports, and the widespread presence of DRIL may contribute to visual dysfunction in treatment-naïve diabetic eyes.

#### EZ disruption

It is indisputable that the retinal photoreceptor layer can be accurately evaluated by examining the integrity of the EZ using OCT, which corresponds to the inner segment of photoreceptors, indicating that EZ integrity has been shown to be strongly correlated with visual acuity^28^. Although the mechanism of photoreceptor degeneration is not yet well defined, several processes may lead to photoreceptor damage. Destructed BRB associated with DM may induce extravasation of blood components and inflammatory cell infiltration, causing morphological abnormalities in the macula^29^. Uji et al. reported that HF is considered a lipoprotein, a precursor of hard exudates, which was incorporated by photoreceptors or macrophages, leading to poor visual acuity^30^. Murakami et al. suggested that incomplete EZ often exists under the cyst space, and that the cyst space of the outer plexiform layer may contribute to photoreceptor damage in DME^31^. In our study, EZ disruption was found in 10.9% of all DM patients, 10.4% in NPDR and 54.2% in PDR, which occurred more frequently in the advanced stages of the disease. Our study also confirmed that the patients showing high frequency of EZ disruption had significantly poorer visual acuity.

#### FAZ circularity

DR is primarily a disease of the retinal vasculature, and OCTA provides valuable information about vascular conditions not available with structural OCT. FAZ is a specialized area of the retina containing the highest density of cone photoreceptors and high oxygen consumption^32^. As the FAZ of the healthy eye usually exhibits a circular or elliptical shape, deviations from this shape are common in vascular pathology^33,34^. Balaratnasingam and colleagues used OCTA to reveal that FAZ area correlates with visual acuity in patients with DR, and proposed FAZ as a biomarker of visual function^27^. FAZ circularity is defined as the ratio of the FAZ boundary to the perimeter of a circle of equal area^33^. Krawitz and colleagues found an association between the disrupted circularity and DR severity, but reported no correlation with visual acuity^35^. On the other hand, Tang et al. demonstrated that FAZ area was not a sensitive marker that correlates with central vision, while decreased FAZ circularity is associated with impaired visual function^36^. In our study, we showed a correlation between visual acuity and FAZ circularity, in which eyes with poor visual acuity had a more irregular shape of FAZ. Alipour et al. explained that studies using the digital curvelet transform revealed that the FAZ of DR deviated from the mildly wavy boundaries found in healthy controls, which became apparent in severely diseased eyes^37^. Defects in the FAZ margin are mainly due to capillary loss and vascular remodeling. The FAZ circularity index allows quantification of disruption of the terminal capillaries in the fovea, which may be a more relevant measure of visual acuity. In addition, macular edema can result in decreased vascular elasticity due to mechanical stretching that results in blockage of blood vessels^38^, and can also change FAZ contours^39^. Therefore, assessing both the size and shape of FAZ is probably important for detecting pathological alteration of the macula in DR.

### Changes in outer and inner retina

In summary, our study showed that the following 3 OCT-based factors could play important roles in impaired visual function in diabetic eyes: length of DRIL, EZ disruption, and FAZ circularity. In addition, these factors had a significant correlation with each other in treatment-naïve diabetic eyes. However, there is a lack of information on how these factors correlate with each other. This study further discusses three categories: (1) DRIL and FAZ circularity, (2) DRIL and EZ disruption, and (3) EZ disruption and FAZ circularity.

#### DRIL and FAZ circularity

Recent studies suggest that DM eye degeneration can be caused by two different states: vasculopathy and neuropathy^40^. NDR and mild NPDR have demonstrated thinning of RNFL due to changes in microcirculation^41^. The metabolic and oxidative stresses of diabetes lead to increased sensitivity of ganglion cells, leading to neuronal loss^42^. After that, DRIL occurs with the severity of retinopathy, which may be negatively correlated with FAZ circularity in SPC. In fact, the frequency of DRIL and FAZ circularity found in this study was significantly high as DR severity increased. Taken together, the relationship between DRIL and FAZ circularity could be associated with both neurovascular damages during DR progression, although segmentation errors in OCTA might exhibit.

#### DRIL and EZ disruption

The sub-analysis of this study showed correlation with DRIL and EZ disruption, suggesting that the extent of DRIL may also be involved in the retinal outer layer in DR. It remains unknown how different cell types die during DR progression. DR is associated with loss of pericytes and endothelial cells in the retinal vasculature, leading to loss of the capillary and BRB^43^. The deep capillary plexus (DCP) including the INL and the OPL are probably important for nutrition to photoreceptor synapses, as the retinal circulation is responsible for approximately 15% of the oxygen supply to photoreceptor inner segments in the darkness^44^. Therefore, photoreceptor cells in the presence of DRIL may be susceptible to ischemic insults in DCP. The presence of DRIL indicates an anatomical disruption of visual transduction pathways in the medial retina and may interfere with the transmission of visual information from photoreceptors to ganglion cells. The anatomical changes observed in this OCT may cause the association with DRIL and EZ disruption.

#### EZ disruption and FAZ circularity

It has also been shown that diabetes is associated with increased loss of retinal neurons^45^. Retinal glial cells including astrocytes, Müller cells and microglia are involved in structural support and maintenance of homeostasis in the retina^46^. Under hyperglycemic stress, microglia are activated, various cytokines are secreted, and astrocytes and Müller cells are involved with the amplification of the inflammatory response by the production of proinflammatory cytokines^47^. Previous studies have reported that FAZ measurements of both SCP and DCP correlate with EZ and IZ disruption, and photoreceptor morphology and integrity are susceptible to macular ischemia^48^. Long-term or more severe macular ischemia not only contributes to the onset of macular edema, but may also cause retinal atrophy due to photoreceptor damage.

It remains open to debate whether diabetic retinal neuropathy is the cause or effect of microangiopathy under chronic hyperglycemia. Barber proposed two hypotheses to explain the relationship between neurodegenerative and microangiopathy changes^49^. First, loss of BRB integrity manifests itself as increased vascular permeability, leading to edema and neuronal loss due to uncontrolled extracellular fluid composition in the retina. Alternatively, DM directly affects metabolism in the neural retina, increasing apoptosis of cells in the neural retina causing a breakdown of the BRB. Despite the close relationship between neurodegeneration and microangiopathy, the progression of DR is ultimately irreversible when untreated. To protect the eyesight of diabetic patients, it is necessary to find ways to prevent the gradual loss of neurons in the retina. Further studies are required to prove the validated association between OCT-based retinal structural abnormalities and visual function in diabetic eyes.

## Limitations

We are aware of some limitations of this study. First, this report includes a retrospective design and a limited number of subjects. A larger sample size in prospective studies is important to reconfirm the results. Second, it was impossible to investigate the VD of the DCP, as the OCTA parameters automatically calculated in AngioPlex relate only to SCP. However, this technique produces projection artifacts in most cases, so SCP analysis is more accurate than DCP one^50^. Third, if the capillary flow rate detected by the OCTA algorithm is below a threshold, the terminal capillaries may not be fully visible. Therefore, measurements of FAZ area and VD may be affected in selected cases. Fourth, demographics such as age, duration of diabetes, HbA1c levels, and hypertension were not used in the regression analysis. This needs to ensure a larger sample size and will reveal a correlation with vision loss. Fifth, the biomorphometry performed in this study utilized data from a single visit. Therefore, it is impossible to use this result to judge the risk of disease progression or to predict response to treatment for DR. Further studies are needed on the natural history of DR and the correlation between post-treatment OCT-based factors and visual acuity. Sixth, we could not examine the degree of pathological alteration and visual impairment in the outer and inner retinas confirmed by OCT images. We need to secure more samples in the future to investigate how parafoveal signaling is related to the degree of visual impairment. Seventh, it was impossible to examine OCT-based variables that correlate with visual acuity in each severity grade of DR, since the subjects were limited to treatment-naïve eyes. Further case collection is required to perform multivariate analysis in each group.

## Conclusions

The length of DRIL, length of EZ disruption, and FAZ circularity measured by OCT were identified as candidate factors for visual impairment in treatment-naïve diabetic eyes.

## Materials and methods

### Patients

This study was a retrospective observational study of patients who visited Teine Keijinkai Hospital from May 2019 to July 2020. The research plan of this study in compliance with the principles of the Helsinki Declaration was approved by Medical Ethics Committee in Teine Keijinkai Hospital (Registration number: 2-020041-00). Written informed consent from each patient was waived because this is a retrospective observational study. Type 2 DM patients and non-DM controls in the Japanese population were included in this study. All type 2 DM patients have been confirmed by a diabetic specialist in Teine Keijinkai Hospital General Internal Medicine and Infectious Diseases Internal Medicine Department or a nearby physician. All subjects underwent a comprehensive ophthalmic examination including best-corrected visual acuity converted to logarithm of the minimum angle of resolution (logMAR), Goldmann applanation tonometry, biomicroscopy of the anterior segment using a slit lamp examination, ophthalmoscopy of the posterior segment with dilated pupils, and axial length measured using optical biometry (IOL Master Zeiss; Jena, Germany) and SD-OCT (Cirrus HD-OCT, Carl Zeiss Meditec, Dublin, CA). Exclusion criteria were eyes with spherical power greater than -5 diopters, cylindrical astigmatism with cylinder power greater than 3 diopters, ocular axial length being greater than 26 mm, cataracts or vitreous hemorrhages, other fundus diseases, and a history of other fundus diseases. Moreover, eyes with a history of previous topical treatments including retinal laser photocoagulation, local vascular endothelial growth factor (VEGF), and/or triamcinolone acetonide injection, or ocular surgeries including vitrectomy or cataract surgery were excluded. Funduscopic examination including the periphery of the retina was performed with a pharmacologically dilated pupil, and the severity of retinopathy was classified into three major grades based on the International Clinical Diabetic Retinopathy and Diabetic Macula Edema Disease Severity Scales^4^: no DR; mild/moderate/severe NPDR; and PDR. Systemic characteristics of diabetic patients were recorded: duration of diabetes, HbA1c, HT, SBP and DBP, eGFR, DL, and TC.

### OCT and OCTA image acquisition

The AngioPlex OCTA software featured an OCT microangiography algorithm that incorporated differences in both phase and intensity information, and a FastTrac retinal tracking system to reduce motion artifacts^51^. OCT imaging used three Scan acquisition options: Macular Cube Scan 200 × 200, single horizontal HD 1 Line 100 × (100 × averaged), and Angiography Scan 6 × 6. They were obtained by an experienced technician and only scans with a signal strength of more than 7/10 (out of 10) were evaluated. To avoid fixation errors, all scans were focused on the fovea and the segmentation errors were corrected manually. Each measurement was independently evaluated by two masked investigators (H.E. and H.T.). When there was disagreement, the evaluation was discussed to reach a consensus.

### OCT-based factors

**Figure 1:**
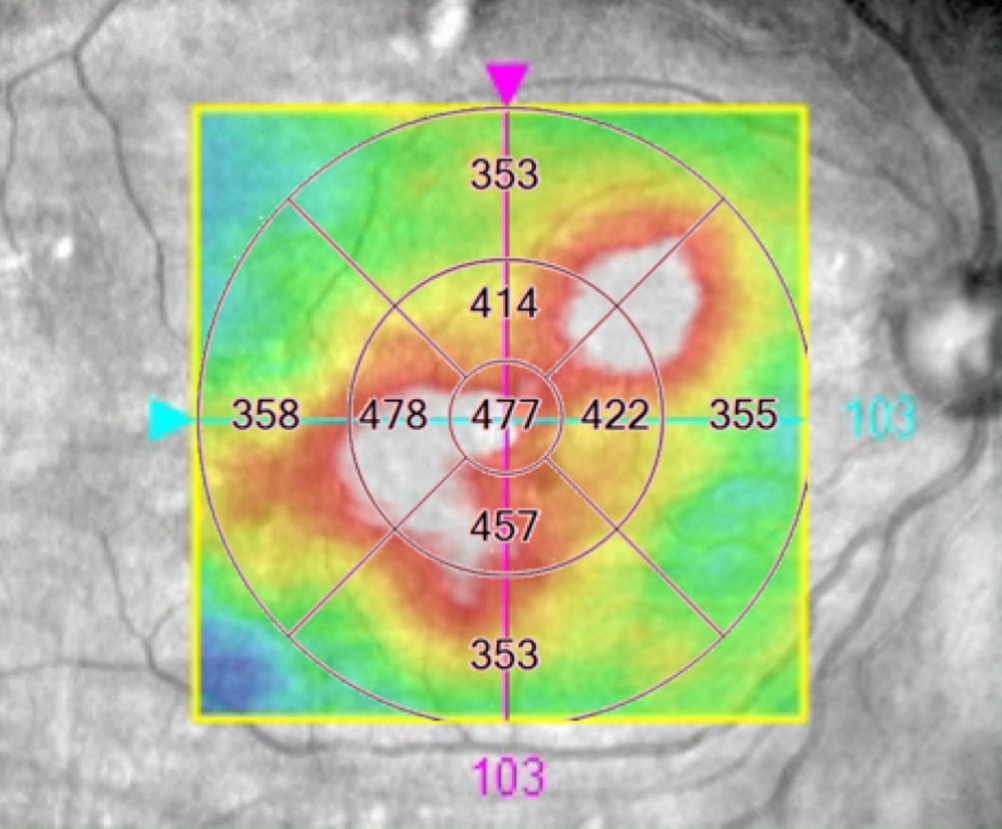
Optical coherence tomography (OCT) C-scan image. The Early Treatment Diabetic Retinopathy Study (ETDRS) 9 grid in 6 × 6 mm scan. The central retinal thickness (CRT) in the central subfield is automatically calculated by the software. In this study, AngioPlex OCT angiography (software version 11.0.0.20046, https://ww.zeiss.com/meditec/int/home.html) was used.

**Figure 2:**
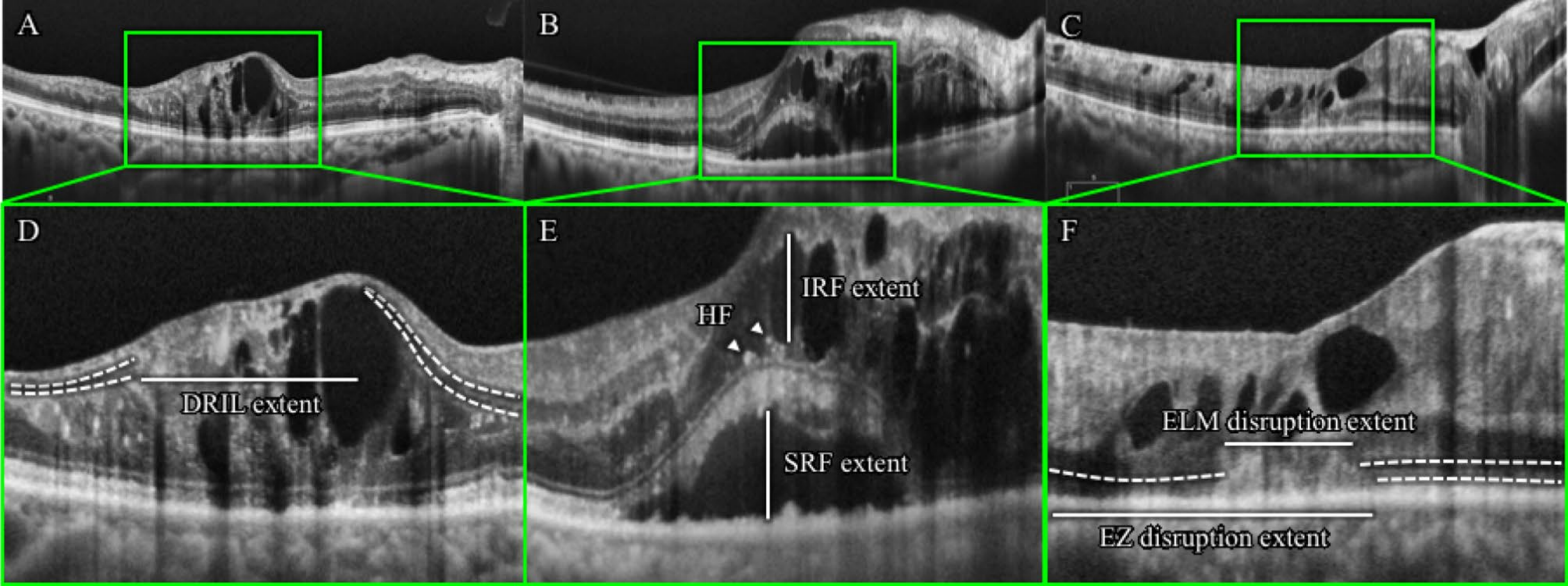
Optical coherence tomography (OCT) B-scan images. (**A**,**D**) The representative image in which disorganization of retinal inner layer (DRIL) existed is shown. The dashed line shows the range where the ganglion cell layer (GCL)/inner plexiform layer (IPL)-inner nuclear layer (INL) and INL-outer plexiform layer (OPL) interface could be confirmed. (**B**,**E**) A representative image in which intraretinal fluid (IRF), subretinal fluid (SRF), and hyperreflective foci (HF) existed is shown. (**C**,**F**) A representative image in which external limiting membrane (ELM) and ellipsoid zone (EZ) disruption existed is shown. The dashed line shows the range where ELM and EZ could be confirmed. Measurement of each lesion was measured manually using a caliper tool mounted on the instrument. In this study, AngioPlex OCT angiography (software version 11.0.0.20046, https://www.zeiss.com/meditec/int/home.html) was used.

The presence of several morphological features was evaluated from B-scan and C-scan images (Figs. 1 and 2); CRT, DRIL, HF, IRF, SRF, ELM, and EZ. Quantitative assessment of CRT was obtained from the central subfield of the Early Treatment Diabetic Retinopathy Study (ETDRS) grid with automated measurements (Fig. 1). Measurement of each lesion was measured manually using a caliper tool mounted on the instrument. DRIL was defined as a horizontal range where the boundaries of the GCL-IPL complex, INL, and OPL could not be identified (Fig. 2A,D). HF was counted manually in each scan (Fig. 2B,E). To exclude hard exudates and microaneurysms from the analysis, we defined them as having the following morphological features: (1) reflectivity similar to that of nerve fiber layer; (2) absence of back-shadowing; and (3) < 30 μm diameter. HF was divided into two groups based on their locations in the retinal layers: the inner retinal layer was defined as the layer between the inner limiting membrane (ILM) and the lower boundary of the outer plexiform layer, and the outer retinal layer was defined as the layer between the outer nuclear layer and the retinal pigment epithelium (RPE)/Bruch's membrane boundaries. IRF was defined as the vertical extent of the hypo-reflection space observed in the outer nuclear layer, and when multiple IRF were present, the IRF closest to the center was selected (Fig. 2B,E). SRF was defined as the vertical distance in the largest hypo-reflective space between the sensory retina and the RPE inner edge (Fig. 2B,E). The extent of ELM and EZ destruction was defined as the horizontal extent of the obscured line (Fig. 2C,F). The extent of DRIL lesions, the number of HF, the presence of IRF and SRF, the extent of ELM and EZ disruption were evaluated in a 1500-μm wide region of the B-scan image centered on the fovea (Fig. 2).

### OCTA parameters

**Figure 3:**
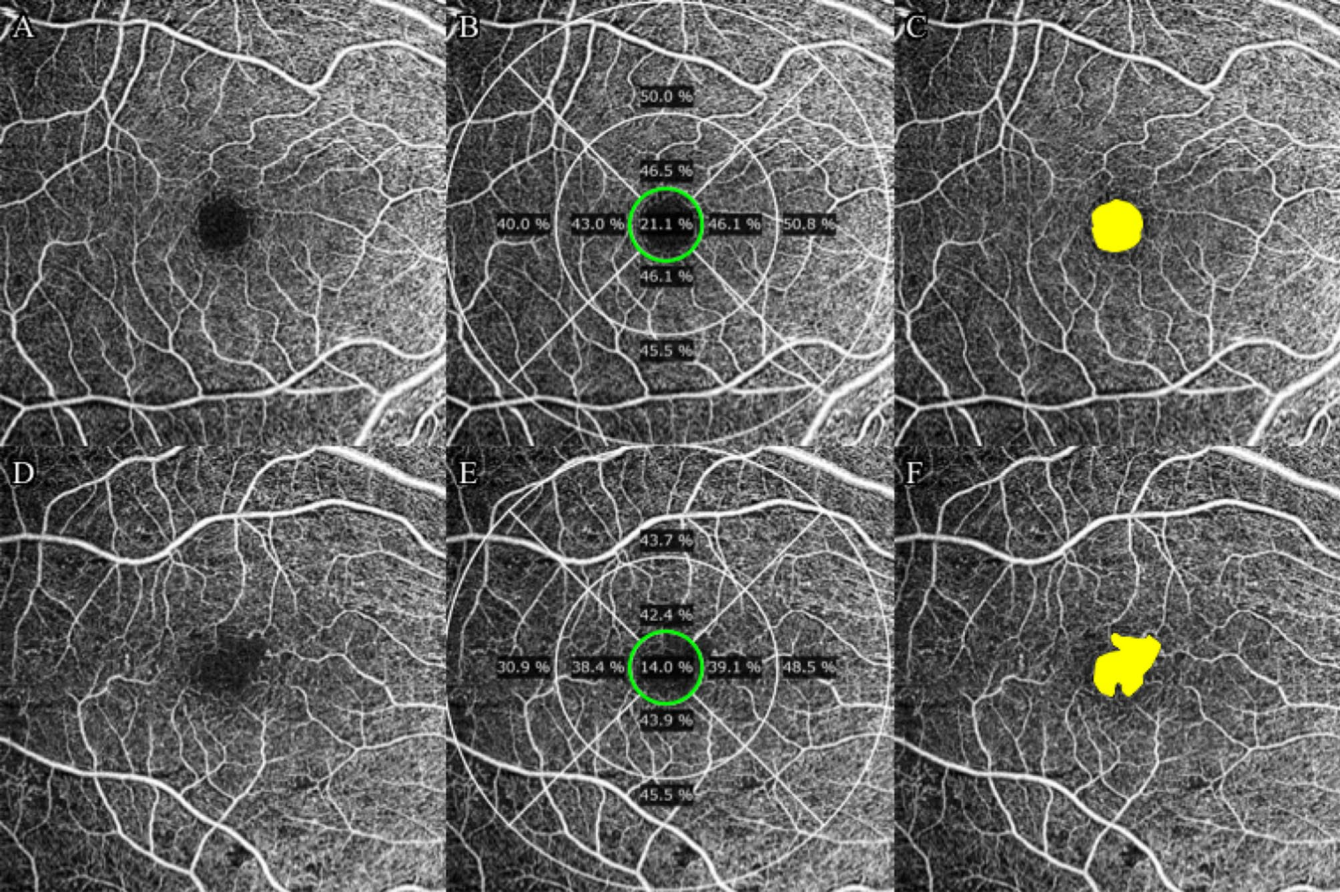
Superficial capillary plexus (SCP) en face images of optical coherence tomography angiography (OCTA). The Early Treatment Diabetic Retinopathy Study (ETDRS) 9 grid in 6 × 6 mm scan. (**A**,**D**) Shows SCP retinal vessels in control and nonproliferative diabetic retinopathy eyes. (**B**,**E**) Vessel density of SCP in central subfield (green ring) is calculated automatically by the software. (**C**,**F**) Foveal avascular zone (FAZ) area and circularity (yellow area) are automatically calculated by the software. In this study, AngioPlex OCT angiography (software version 11.0.0.20046, https://www.zeiss.com/meditec/int/home.html) was used.

OCTA images of the SCP were generated using an automated software algorithm (Fig. 3A,D). The upper boundary of the SCP slab is the ILM and the lower boundary is an approximation of the IPL inferred by the following Eq. (1):

1 $${\text{Z}}_{{{\text{IPL}}}} \, = \,{\text{Z}}_{{{\text{ILM}}}} \, + \,70\% \times {\text{T}}_{{{\text{ILM}} - {\text{OPL}}}} ,$$

where Z, T, and OPL indicate the boundary location, thickness, and outer plexiform layer, respectively. Several quantitative parameters were evaluated from OCTA images automatically generated from Angiography scan 6 × 6 mm slabs according to previous reports: VD, FAZ area, FAZ circularity^52^. VD was defined as the total area of perfused vasculature per unit area in the measurement area, determined by summing the number of pixels with flow signal and dividing the total number by the number of pixels in the study area. Quantitative assessment of VD was obtained from the central (1 mm circle) of the ETDRS grid with automated measurements (Fig. 3B,E). FAZ area was defined as the area included within the FAZ boundary (Fig. 3C,F). FAZ circularity indicates how close the FAZ boundary is to a circle, with a range from 0 to 1, with a value of 1 meaning that FAZ is a complete circle.

### Statistical analysis

All data were expressed as mean ± standard deviation. Categorical variables used Chi-square test and Fisher's exact test. Differences in various characteristics between DM patients and controls were compared using the Mann–Whitney U test. Comparison of clinical factors, OCT/OCTA-based factors between DM patients and controls used the Mann–Whitney *U* test. Comparison of clinical factors, OCT/OCTA-based factors at various DR stages used the Kruskal–Wallis and Steel–Dwass tests. Multivariate analysis was performed to identify factors significantly associated with visual acuity. Statistical analysis was performed using commercially available statistical software SPSS version 21 (IBM Corporation, New York, USA). *P* values < 0.05 were considered to indicate statistical significance.

## Data Availability

The dataset generated and analyzed in this study are available from the corresponding author upon reasonable request.
